# A reliability assessment of the basic erosive wear examination and the tooth wear evaluation system 2.0 utilizing intraoral scan data

**DOI:** 10.1371/journal.pone.0351330

**Published:** 2026-06-12

**Authors:** Maria Lorens, Iwona Tomaszewska

**Affiliations:** 1 Tomaszewska Stomatologia, Cracow, Poland; 2 Department of Medical Education, Centre of Innovative Medical Education, Jagiellonian University Medical College, Cracow, Poland; Universidade Federal de Pelotas, BRAZIL

## Abstract

**Objectives:**

This study assessed the reliability and clinical applicability of two tooth wear screening indices—Basic Erosive Wear Examination (BEWE) and the Tooth Wear Screening module of the Tooth Wear Evaluation System 2.0 (TWES 2.0)—using intraoral scans.

**Materials and methods:**

A total of 246 anonymized intraoral scans from adult patients were independently evaluated by two calibrated examiners. Examiner calibration was performed prior to the study using a representative set of intraoral scans. Calibration was repeated until consensus regarding the application of the scoring criteria was achieved before formal data collection. Scores for all sextants were recorded for BEWE and TWES 2.0. Inter-rater agreement was primarily assessed using weighted kappa coefficients, as BEWE and TWES 2.0 are ordinal, numerically coded indices. Wilcoxon signed-rank tests were additionally used to assess systematic directional differences between paired scores. Statistical significance was set at p < 0.05.

**Results:**

BEWE demonstrated good reliability, with weighted kappa values ranging from 0.760 to 0.851 across sextants, 0.868 for the total BEWE score, and an overall weighted kappa of 0.841. TWES 2.0 showed moderate to good reliability, with weighted kappa values ranging from 0.543 to 0.761 across sextants and an overall weighted kappa of 0.715.

**Conclusions:**

Both BEWE and TWES 2.0 are reliable and practical for screening noncarious tooth wear via intraoral scans. BEWE showed slightly higher inter-rater consistency, whereas TWES 2.0 allows more detailed evaluation of occlusal and palatal surfaces. These indices can support standardized monitoring, early detection, and clinical management of tooth wear. Examiner calibration remains essential, particularly for TWES 2.0.

**Clinical trial registration:**

This was a retrospective reliability study and did not involve an interventional clinical trial; therefore, registration was not applicable.

## 1. Introduction

Tooth wear is a multifactorial condition characterized by the non-carious loss of dental hard tissues. It results from three principal mechanisms—erosion, attrition, and abrasion—which may act independently or synergistically [1,2], with erosion often considered the predominant factor [3]. As an irreversible process, tooth wear progressively compromises enamel and dentin, leading to clinical problems such as dentin hypersensitivity, loss of vertical dimension, impaired esthetics [4]. It is also increasingly recognized as a clinically relevant condition requiring early identification and monitoring [5].

The prevalence of tooth wear has increased worldwide and is now frequently observed across all age groups, with a particularly concerning rise among younger patients [6–8]. Epidemiological studies report a wide prevalence range in adult populations, varying from approximately 20% to over 50%, depending on age, diagnostic criteria, and assessment method. While early-stage tooth wear is often asymptomatic, moderate to severe forms have been associated with functional impairments, including loss of occlusal vertical dimension, compromised masticatory efficiency, dentin hypersensitivity, and increased need for complex restorative interventions [9-10]. Importantly, a substantial proportion of younger adults already exhibit early signs of erosive and attritional wear, underscoring the clinical relevance of early detection and preventive screening strategies. In this context, the use of standardized, reproducible screening indices is essential to enable timely diagnosis, risk stratification, and monitoring of disease progression [4,6,8]. This growing burden highlights the need for reliable diagnostic and monitoring methods that can be applied effectively in everyday clinical practice [9].

To promote standardization in the assessment of tooth wear, the 2017 European Consensus Statement recommended three principal indices: the Basic Erosive Wear Examination (BEWE), the Tooth Wear Index (TWI), and the Tooth Wear Evaluation System 2.0 (TWES 2.0) [11]. These tools were identified as valid frameworks for recording the severity and distribution of wear. However, their clinical usefulness differs. Although the Tooth Wear Index (TWI) remains a valuable reference for detailed epidemiological and research purposes, its extensive scoring system and time-consuming application limit its feasibility for rapid screening and routine clinical use [10]**.** For this reason, the present study focused exclusively on BEWE and the Tooth Wear Screening Module of TWES 2.0, which are designed for efficient clinical screening [12-13]**.** By contrast, the BEWE and TWES 2.0 include simplified scoring systems designed to facilitate rapid chairside assessment, making them more practical as screening tools [14,15]. In particular, the Tooth Wear Screening component of TWES 2.0 is especially well-suited for initial examinations, providing a quick overview of tooth wear status that can efficiently guide further diagnostic or preventive steps.

While both BEWE and TWES 2.0 are recommended for clinical assessment of tooth wear, they differ in scope and level of detail. BEWE is a simplified screening index primarily focused on erosive tooth wear, offering high feasibility and ease of use in routine clinical practice, but providing limited information on wear distribution and etiology. In contrast, TWES 2.0 adopts a broader conceptual framework that encompasses erosion, attrition, and abrasion, allowing for a more detailed surface-specific assessment, particularly on occlusal and palatal surfaces. This increased diagnostic detail, however, may come at the cost of greater examiner dependence and potentially reduced reproducibility. Comparing these indices when applied to intraoral scans may therefore provide valuable insights into their relative reliability and practical applicability in digital workflows.

Although several indices are available for assessing tooth wear, BEWE and the Tooth Wear Screening Module of TWES 2.0 were selected for this study due to their feasibility for routine clinical use and compatibility with digital workflows. Both indices are designed for rapid screening and rely on simplified, ordinal scoring systems that can be readily applied to intraoral scans. In contrast, the Tooth Wear Index (TWI), while valuable for detailed epidemiological assessment, requires extensive surface-level scoring and is less suited to time-efficient screening or digital assessment. Previous studies have also highlighted the practical advantages of BEWE and TWES 2.0 when applied in a digital context, supporting their use as screening tools in contemporary dental practice [13-16].

Previous studies have evaluated tooth wear indices using clinical examinations, dental casts, and, in selected reports, digital models [13-16]. However, available evidence remains heterogeneous, and data on the inter-rater reliability of BEWE and TWES 2.0 when applied exclusively to intraoral scan–derived digital models are still limited. In particular, it remains unclear whether these screening indices maintain consistent examiner agreement when assessments rely solely on three-dimensional digital models rather than direct clinical inspection [17,16]. Given the increasing use of intraoral scanners in routine dental workflows, establishing the reproducibility of BEWE and TWES 2.0 in a fully digital context is clinically meaningful.

The objective of this study was to evaluate and compare the inter-rater reliability of the Basic Erosive Wear Examination (BEWE) and the Tooth Wear Screening Module of the Tooth Wear Evaluation System 2.0 (TWES 2.0) when applied to intraoral scan–derived digital models. Specifically, the study aimed to assess examiner agreement across sextants using standardized reliability metrics.

Two null hypotheses were formulated. First, it was hypothesized that no systematic differences would exist between the scores assigned by the two examiners for either BEWE or TWES 2.0 when applied to intraoral scans. Second, it was hypothesized that both indices would demonstrate comparable levels of inter-rater reliability when applied to intraoral scans.

## 2. Materials and methods

### 2.1. Study design and ethical considerations

This retrospective study analyzed anonymized intraoral scans to evaluate the reliability and clinical applicability of two tooth wear screening indices: the Basic Erosive Wear Examination (BEWE) and the Tooth Wear Screening module of the Tooth Wear Evaluation System 2.0 (TWES 2.0). The study was conducted in accordance with the Declaration of Helsinki. Ethical approval was obtained from the Bioethics Committee at the District Medical Chamber in Kraków, Poland (approval number: L.dz.OIL/KBL/22/2025, issued on 27 May 2025). Due to the retrospective and anonymized nature of the data, individual patient consent was not required; written permission to use the scans was obtained from all three participating dental clinics.

This study evaluated whether the BEWE and the TWES 2.0 Screening Module can be applied consistently to intraoral scan–derived digital models, focusing on the reproducibility of scoring rather than diagnostic accuracy.

#### 2.1.1. Reporting guidelines.

This study was reported with consideration of the STARD 2015 recommendations for diagnostic studies, where applicable. Because the present study was designed as an inter-rater reliability study of two tooth wear screening indices applied to intraoral scan-derived digital models, rather than a diagnostic accuracy study against a reference standard, STARD items related to a reference standard and diagnostic accuracy estimates were considered not applicable.

### 2.2. Description of indices

#### 2.2.1. BEWE.

The Basic Erosive Wear Examination (BEWE), is a standardized and widely applied screening tool for erosive tooth wear [12,18-19]. The dentition is divided into sextants, and in each sextant the surface with the most severe lesion is recorded. Third molars are excluded. The sextants were defined as follows: Sextant 1 (maxillary right posterior: teeth 14–17), Sextant 2 (maxillary anterior: teeth 13–23), Sextant 3 (maxillary left posterior: teeth 24–27), Sextant 4 (mandibular left posterior: teeth 34–37), Sextant 5 (mandibular anterior: teeth 33–43), and Sextant 6 (mandibular right posterior: teeth 44–47). Surfaces are scored on a four-point ordinal scale according to the following criteria:

Score 0 – No evidence of erosive tooth wear.
Score 1 – Initial loss of surface texture, with early enamel softening but without distinct tissue loss.
Score 2 – Distinct hard tissue loss involving less than 50% of the surface area.
Score 3 – Hard tissue loss involving more than 50% of the surface area.

The cumulative BEWE score is obtained by summing the sextant scores across the full dentition. This sum can then be used to stratify patients into risk categories (none, low, medium, or high risk), which are linked to clinical recommendations for monitoring and management. The system is designed to be simple, reproducible, and feasible for rapid chairside application, while still allowing clinicians to make informed decisions about preventive or restorative needs.

For the purposes of the present study, however, BEWE scores were recorded and analyzed separately for each sextant, with each sextant constituting an individual unit of analysis.

#### 2.2.2. Tooth wear evaluation system 2.0 (TWES 2.0).

The Tooth Wear Evaluation System 2.0 (TWES 2.0) is a standardized framework for assessing non-carious tooth wear caused by erosion, attrition, and abrasion [14,15]. TWES 2.0 consists of two modules: the Tooth Wear Screening Module, intended for rapid clinical or epidemiological assessments, and the Tooth Wear Status Module, which allows a more detailed evaluation for treatment planning and comprehensive documentation. While the Status module enables extended analysis of individual surfaces and severity, in this study we focused exclusively on the Screening Module, which facilitates quick, reliable assessment in routine practice and large-scale studies [15].

In the Tooth Wear Screening Module, the dentition is divided into sextants, and the most severely affected surface within each sextant is recorded. Palatal surfaces in Sextant 2 receive additional evaluation to ensure thorough assessment of this often-affected area. Scoring uses two separate five-point ordinal scales:

1. Occlusal and incisal surfaces:
  Grade 0: No visible wear
  Grade 1: Wear limited to enamel
  Grade 2: Exposed dentin with ≤ 1/3 loss of clinical crown height
  Grade 3: Loss of crown height > 1/3 but < 2/3
  Grade 4: Loss of crown height ≥ 2/3
2. Non-occlusal surfaces (buccal and lingual):
  Grade 0: No visible wear
  Grade 1: Wear limited to enamel
  Grade 2: Exposed dentin affecting < 50% of the surface
  Grade 3: Exposed dentin affecting ≥ 50% of the surface
  Grade 4: Complete enamel loss or pulp exposure

Scores are recorded independently for each sextant, without summing across sextants. Clinical recommendations are guided by severity: Grades 0–1 indicate routine monitoring, Grade 2 may prompt further evaluation, and Grades 3–4 require a comprehensive Status module assessment. This modular approach ensures that the Screening Module remains efficient and practical, while the Status module can be applied when more detailed clinical assessment is needed.

Accordingly, in this study, each sextant score constituted a separate data point for statistical analysis.

### 2.3. Data collection and examiner calibration

A total of 246 intraoral scans from unique adult patients (≥18 years) were included in the study. Due to the retrospective and anonymized nature of the dataset, limited demographic information was available. Sex was available for descriptive purposes only, with 129 female, 66 male, and 51 unknown or unreported cases. No additional patient-level information, including exact age, medical history, or behavioral factors, was available. Sex was not used as an analytical variable and did not affect the reliability analyses. The anonymized intraoral scan data were first accessed for research purposes on 12/05/2025. Scans were acquired in two clinics using the Medit i500 scanner (Medit Corp., Seoul, South Korea) and in one clinic using the iTero Element 2 scanner (Align Technology, San Jose, CA, USA). Due to the retrospective nature of the dataset, the exact distribution of scans between scanner systems was not recorded. Both systems provide high-resolution full-arch digital models suitable for qualitative tooth wear assessment and digital wear analysis [20,16]; therefore, scans were pooled for analysis.

All scans were exported in a high-resolution mesh format (.stl or.ply files), depending on the format provided by the clinical centers, and were analyzed using standard dental imaging software capable of rendering full-arch three-dimensional digital models. To ensure consistency, all scans were assessed under identical conditions, using the same type of display monitor and lighting. All intraoral scans were analyzed using Medit Link software (version 3.3.6; Medit Corp., Seoul, South Korea), which was used for storage, retrieval, and visualization of three-dimensional digital models. Both STL and PLY file formats were directly supported by the software and were visualized without file conversion.

Both examiners used the same software version throughout the study.

Three-dimensional models were assessed using standard visualization functions provided by the software, including free rotation and zooming. No sectioning tools, slicing functions, or automated measurement features were applied. All evaluations were based on visual inspection of the digital models.

To ensure consistency, both examiners worked under identical software environments, using the same software version, display monitor type, and ambient lighting conditions.

Intraoral scans were included if they originated from adult patients (≥18 years), the digital model contained at least 14 teeth, and at least 50% of the tooth surface area was free from extensive restorations, prosthetic reconstructions, or large fillings. Scans presenting major artifacts, incomplete arches, or extensive restorative coverage preventing reliable assessment were excluded from the analysis.

Two independent examiners, trained and familiarized with the scoring protocols, evaluated each scan using both indices (BEWE and the TWES 2.0 Tooth Wear Screening module only). Both examiners had more than five years of clinical experience in restorative and preventive dentistry.

Prior to formal data collection, a calibration process was conducted using a set of intraoral scans that were not included in the final analysis. The calibration set was selected to represent a broad spectrum of tooth wear presentations, including teeth without visible wear and teeth with mild, moderate, and advanced wear lesions across different sextants and surfaces. The term “representative” therefore referred to variation in wear severity and anatomical location. Calibration was repeated until consensus regarding the application of BEWE and TWES 2.0 scoring criteria was achieved.

Each sextant was scored independently by both raters, and the results were recorded separately to allow assessment of interobserver reliability.

Missing sextant-level data resulted from localized scan limitations, such as incomplete capture of specific sextants, artifacts, or extensive restorative coverage preventing reliable scoring. Only paired sextant observations evaluated by both examiners were included in inter-rater reliability analyses, which explains minor variations in sample size across sextants.

Each participant was scanned once using a single intraoral scanner (either Medit i500 or iTero Element 2). Each intraoral scan was independently evaluated by two examiners using both screening indices (BEWE and the TWES 2.0 Tooth Wear Screening Module). For BEWE, one score per sextant was recorded, resulting in six BEWE scores per participant per examiner. For TWES 2.0, one score per sextant was recorded for all sextants, with an additional score recorded for the palatal surface in Sextant 2, in accordance with the screening protocol. Accordingly, for each participant, each examiner generated six BEWE scores and seven TWES 2.0 scores. The additional palatal surface score in Sextant 2 was analyzed as a distinct data point within the TWES 2.0 dataset and did not affect the BEWE scoring scheme. All scores were treated as separate data points for statistical analysis.

As intraoral scan–derived mesh files do not contain color or material-specific information, identification of enamel and dentin involvement was based on morphological features of tooth wear, in accordance with the original clinical definitions of BEWE and TWES 2.0. In cases of uncertainty, surfaces were not scored.

All intraoral scans were evaluated independently by both examiners. The examiners were blinded to each other’s scores and did not have access to their previously recorded scores during the scoring phase. Scoring was performed without knowledge of patient identifiers or clinical information beyond the digital models. To minimize potential order effects, scans were assessed in a randomized order for each examiner. Once recorded, scores were not revised or modified. All evaluations were completed independently, without discussion between examiners during the scoring phase.

Scanner type was not included as an analytical variable, as the primary aim of the study was to assess inter-rater reliability of scoring rather than to compare measurement systems. Both scanners used in this study (Medit i500 and iTero Element 2) are high-resolution intraoral scanners widely applied in clinical practice and considered suitable for qualitative assessment of tooth wear. However, potential variability related to scanner type cannot be completely excluded.

### 2.4. Sample size consideration

This retrospective reliability study included 246 intraoral scans, generating multiple sextant-level observations assessed independently by two examiners. The resulting sample size is comparable to or exceeds that of previously published reliability studies evaluating tooth wear indices and digital assessment methods. Accordingly, the sample size was considered adequate to detect meaningful levels of agreement and to minimize the risk of type II error.

To further contextualize the adequacy of the available sample size, a supplementary sample size assessment was performed in R software (version 4.4.3) using the *irr* package and the N2.cohen.kappa() function. Because no dedicated implementation for weighted kappa sample size estimation was available in the applied framework, the calculations were based on Cohen’s kappa for two raters, using the observed marginal category distributions for each sextant and the observed agreement coefficients as anticipated agreement levels. The null hypothesis agreement level was set at κ₀ = 0.40, corresponding to moderate agreement, with a two-sided α = 0.05. Required sample sizes were calculated for 80%, 90%, and 95% statistical power.

For BEWE, the observed number of paired sextant-level observations (N = 238–246 across sextants) exceeded the required sample size for 95% power in all sextants (required N = 49–75), indicating that the study was well powered for BEWE agreement analyses. For TWES 2.0, the situation was more heterogeneous: adequate power was observed for some sextants with stronger agreement (particularly Sextant 2 palatal and Sextant 4), whereas for other sextants the required sample size exceeded the observed number of paired observations. These findings suggest that the study was clearly adequately powered for BEWE and informative for TWES 2.0, although some TWES sextant-specific estimates should be interpreted with greater caution. Overall, these findings indicate that the study was sufficiently powered to detect meaningful levels of inter-rater agreement, particularly for BEWE, while TWES 2.0 estimates should be interpreted with some caution in selected sextants.

### 2.5. Statistical analysis

Because BEWE and TWES 2.0 scores are ordinal, although numerically coded, descriptive statistics were interpreted primarily using medians and interquartile ranges (Q1-Q3). Data distribution was visualized using boxplots, with the median indicated by the line within the box and outliers represented as smaller points.

Inter-rater agreement between examiners was assessed using weighted kappa coefficients with 95% confidence intervals, as BEWE and TWES 2.0 are ordinal scoring systems. Weighted kappa coefficients were used as the primary measure of inter-rater agreement. Wilcoxon signed-rank tests were additionally performed as supplementary analyses to explore potential systematic directional tendencies between paired ratings and were not interpreted as measures of agreement.

All statistical analyses were conducted using R software (version 4.4).

Each index was analyzed separately; no cross-scale conversion or direct numerical comparison between BEWE and TWES 2.0 scores was performed. In addition to sextant-level analyses, overall weighted kappa coefficients were calculated to provide a global summary of inter-rater agreement across all analyzed observations.

## 3. Results

### 3.1. BEWE

For each sextant, analyses were based on paired observations from both raters; therefore, the reported sample size (N) represents the number of sextants evaluated by both examiners.

Table 1 presents a comparison of BEWE scores between Rater 1 and Rater 2 across all sextants and the total score. Median values and interquartile ranges were highly similar or identical between raters, indicating comparable overall score distributions. Although Wilcoxon signed-rank tests detected small but statistically significant directional asymmetries in paired observations across several sextants and for the total BEWE score, these findings reflect minor systematic tendencies in scoring rather than clinically meaningful disagreement. Inter-rater reliability was therefore assessed primarily using weighted kappa coefficients.

**Table 1:** Comparison of BEWE sextant scores between raters.

Variable	Parameter	Overall (paired observations)	Rater 1 (N = 246)	Rater 2 (N = 246)	Paired difference	test	p-value
Sextant 1	N	241	241	241	241	Wilcoxon	**0.0039 (S)**
	Median (Q1-Q3)	2 (1 – 2)	2 (1 – 2)	2 (1 – 2)	0 (0 − 0)		
Sextant 2	N	246	246	246	246	Wilcoxon	**0.0066 (S)**
	Median (Q1-Q3)	3 (2 – 3)	3 (2 – 3)	3 (2 – 3)	0 (0 − 0)		
Sextant 3	N	238	238	238	238	Wilcoxon	**<0.001 (S)**
	Median (Q1-Q3)	2 (1 – 2)	2 (2 – 2)	2 (1 – 2)	0 (0 − 0)		
Sextant 4	N	245	245	245	245	Wilcoxon	**0.0137 (S)**
	Median (Q1-Q3)	2 (2 – 2)	2 (2 – 3)	2 (2 – 2)	0 (0 − 0)		
Sextant 5	N	246	246	246	246	Wilcoxon	**<0.001 (S)**
	Median (Q1-Q3)	3 (2 – 3)	3 (2 – 3)	3 (2 – 3)	0 (0 − 0)		
Sextant 6	N	238	238	238	238	Wilcoxon	**<0.001 (S)**
	Median (Q1-Q3)	2 (2 – 2)	2 (2 – 3)	2 (2 – 2)	0 (0 − 0)		
Total BEWE	N	246	246	246	246	Wilcoxon	**<0.001 (S)**
	Median (Q1-Q3)	13 (11 - 14.25)	13 (12 – 1411 – 15)	13 (12, 11 – 14)	0 (−1 − 0)		

Inter-rater reliability was assessed using weighted kappa coefficients. For BEWE, weighted kappa values ranged from 0.760 to 0.851 across sextants, indicating good agreement between examiners (Table 2). The weighted kappa for the total BEWE score was 0.868, and the overall weighted kappa across sextant-level observations was 0.841. Weighted kappa values were interpreted according to Altman’s criteria [21].

**Table 2:** Inter-rater reliability of BEWE scores assessed using weighted kappa coefficients.

Variable	N	Weighted kappa (95% CI)	Statistic	Weighted kappa p-value
Sextant 1	241	0.802 (0.737–0.867)	12.6	**<0.001**
Sextant 2	246	0.851 (0.804–0.898)	13.4	**<0.001**
Sextant 3	238	0.760 (0.669–0.851)	12.0	**<0.001**
Sextant 4	245	0.850 (0.799–0.901)	13.4	**<0.001**
Sextant 5	246	0.826 (0.754–0.898)	13.1	**<0.001**
Sextant 6	238	0.799 (0.726–0.872)	12.5	**<0.001**
Total BEWE	246	0.868 (0.813–0.923)	11.3	**<0.001**
Overall weighted kappa	1454	0.841 (0.817–0.864)	32.3	**<0.001**

Agreement patterns between raters were generally consistent across sextants, with most discrepancies occurring within adjacent BEWE score categories.

N represents the number of paired sextant-level observations scored independently by both examiners and included in the statistical analyses.

S indicates statistical significance (p < 0.05); NS indicates non-significance.

### 3.2. TWES 2.0 (Screening module)

For each sextant, analyses were based on paired observations from both raters; therefore, the reported sample size (N) represents the number of sextant-level observations evaluated by both examiners. For the TWES 2.0 Screening Module, the palatal surface in Sextant 2 was analyzed as an additional paired observation.

Table 3 presents TWES 2.0 Screening scores across all sextants for Rater 1 and Rater 2. Small but statistically significant differences were observed in most sextants, with Rater 1 generally assigning slightly higher scores.

**Table 3:** Comparison of TWES 2.0 Screening Module sextant scores between raters.

Variable	Parameter	Overall (paired observations)	Rater 1 (N = 246)	Rater 2 (N = 246)	Paired difference	test	p-value
Sextant 1	N	237	237	237	237	Wilcoxon	**0.0243 (S)**
	Median (Q1-Q3)	1 (1 – 1)	1 (1 – 1)	1 (1 – 1)	0 (0 − 0)		
Sextant 2 inc.	N	245	245	245	245	Wilcoxon	**<0.001 (S)**
	Median (Q1-Q3)	2 (1 – 2)	2 (1 – 2)	1 (1 – 2)	0 (0 − 0)		
Sextant 2 pal.	N	244	244	244	244	Wilcoxon	**<0.001 (S)**
	Median (Q1-Q3)	1 (0 - 1)	1 (0 - 1)	1 (0 - 1)	0 (0 − 0)		
Sextant 3	N	234	234	234	234	Wilcoxon	**<0.001 (S)**
	Median (Q1-Q3)	1 (1 – 1)	1 (1 – 1)	1 (1 – 1)	0 (0 − 0)		
Sextant 4	N	243	243	243	243	Wilcoxon	**0.0128 (S)**
	Median (Q1-Q3)	1 (1 – 1)	1 (1 – 1)	1 (1 – 1)	0 (0 − 0)		
Sextant 5	N	246	246	246	246	Wilcoxon	**<0.001 (S)**
	Median (Q1-Q3)	2 (1 – 2)	2 (1.25 - 2)	2 (1 – 2)	0 (0 − 0)		
Sextant 6	N	237	237	237	237	Wilcoxon	**0.0014 (S)**
	Median (Q1-Q3)	1 (1 – 1)	1 (1 – 2)	1 (1 – 1)	0 (0 − 0)		

For TWES 2.0, weighted kappa values ranged from 0.543 (Sextant 1) to 0.761 (Sextant 2 palatal), indicating moderate to good agreement across sextants. The overall weighted kappa for TWES 2.0 was 0.715. Unlike BEWE, TWES 2.0 Screening Module scores were not summed across sextants; therefore, no cumulative total score was calculated.

Agreement patterns between raters were consistent, with most discrepancies occurring within adjacent TWES 2.0 score categories, reflecting overall stability of the screening results.

N represents the number of paired sextant-level observations scored independently by both examiners and included in the statistical analyses.

S indicates statistical significance (p < 0.05); NS indicates non-significance Table 4.

**Table 4:** Inter-rater reliability of TWES 2.0 Screening Module scores assessed using weighted kappa coefficients.

Variable	N	Weighted kappa (95% CI)	Statistic	Weighted kappa p-value
Sextant 1	237	0.543 (0.410–0.676)	8.54	**<0.001**
Sextant 2 inc.	245	0.587 (0.495–0.680)	9.44	**<0.001**
Sextant 2 pal.	244	0.761 (0.694–0.828)	12.05	**<0.001**
Sextant 3	234	0.647 (0.525–0.770)	10.18	**<0.001**
Sextant 4	243	0.677 (0.566–0.788)	10.66	**<0.001**
Sextant 5	246	0.619 (0.520–0.719)	9.97	**<0.001**
Sextant 6	237	0.589 (0.473–0.705)	9.24	**<0.001**
Overall weighted kappa	1686	0.715 (0.684–0.747)	29.8	**<0.001**

## 4. Discussion

The present study assessed inter-rater reliability and patterns of tooth wear using two indices—BEWE and TWES 2.0—applied to intraoral scan-derived digital models. The study focused on reproducibility of scoring rather than diagnostic accuracy against a clinical reference standard. The results provide important insights into the reproducibility and clinical utility of these tools for evaluating noncarious tooth wear.

The good inter-rater reliability observed for BEWE in the present study is consistent with previous clinical investigations, which have demonstrated that BEWE provides reproducible and clinically robust assessments of erosive tooth wear when applied by calibrated examiners [12,18,19,22].

According to commonly accepted benchmarks, the observed weighted kappa values indicate good agreement for BEWE and moderate to good agreement for TWES 2.0 across sextants.

BEWE demonstrated consistently high inter-rater agreement across sextants, confirming its reproducibility when applied to intraoral scan-derived digital models. These findings are consistent with previous studies reporting good examiner agreement for BEWE in clinical settings [12,18-19]. These findings suggest that BEWE is a consistent tool for clinical screening of erosive tooth wear, demonstrating good inter-examiner agreement in the present study. The consistency of BEWE scores confirms its suitability for routine clinical practice and epidemiological studies.

TWES 2.0 demonstrated slightly lower agreement than BEWE, which is consistent with previous reports indicating that more detailed, surface-specific indices may increase examiner dependence [14,15,23,24]. Although these results support moderate to good inter-rater reliability, the power assessment showed that agreement estimates for some TWES sextants were based on less favorable sample size conditions than for BEWE and should therefore be interpreted with somewhat greater caution. Minor variability observed in certain sextants—particularly posterior and palatal surfaces—likely reflects the increased difficulty of grading subtle wear patterns in these regions rather than a systematic bias.

An additional factor that may have influenced inter-rater agreement is the assessment of dentine exposure. Even under clinical conditions, identification of exposed dentine has been shown to have only moderate accuracy when compared with histological validation [25]. This limitation may be further amplified when assessments are based on intraoral scan–derived models without color information. In the context of TWES 2.0, dentine exposure represents a key threshold between grades 1 and 2, and difficulties in its identification can contribute to variability between examiners. A similar, although less clearly defined, effect may also be present in BEWE scoring, where dentine involvement can contribute to higher scores. This aspect should be considered when interpreting the observed differences in reliability between the two indices. This may partially explain the slightly lower agreement observed for TWES 2.0 in the present study.

Although statistically significant differences between raters were observed for BEWE scores across sextants, these differences were generally small and predominantly confined to adjacent score categories. From a clinical perspective, such minor discrepancies are unlikely to result in systematic changes in BEWE risk category classification, which is the primary basis for clinical decision-making and patient management. Therefore, the observed statistical significance should be interpreted with caution, as it does not necessarily indicate clinically meaningful disagreement between examiners.

In the TWES 2.0 Screening Module, inter-rater reliability varied across sextants and surfaces. Higher agreement was observed for anterior and palatal surfaces, particularly in the palatal component of Sextant 2, which demonstrated the highest weighted kappa value. This finding may be explained by the typically more uniform and clearly demarcated wear patterns on palatal surfaces of maxillary anterior teeth, which are commonly affected by erosive processes and therefore easier to identify consistently on digital models.

In contrast, slightly lower weighted kappa values were noted in posterior sextants, likely reflecting the increased morphological complexity of occlusal surfaces and the more subtle transitions between wear grades in these regions. Differences between anterior and posterior regions therefore appear to be related to surface anatomy and visual interpretability rather than systematic examiner bias. Overall, these findings support the robustness of TWES 2.0 for screening purposes, while highlighting that posterior and occlusal surfaces may require particular attention during examiner calibration.

While previous studies have evaluated tooth wear indices using clinical examinations and dental casts, evidence regarding their application to intraoral scan–derived digital models remains limited. The present findings therefore extend existing knowledge by demonstrating that both BEWE and TWES 2.0 can be applied with acceptable reliability in a fully digital assessment environment [13-16].

It should be emphasized that high inter-rater reliability does not necessarily imply diagnostic validity. Therefore, although the present study confirms consistent application of both indices on intraoral scans, further validation against clinical examination is required to establish their accuracy in digital settings.

For BEWE, scores were higher overall, reflecting a broader scoring range and the tool’s sensitivity to more pronounced erosive wear. The distribution of scores confirms BEWE’s suitability for rapid screening, while TWES 2.0 provides detailed surface-specific assessment.

The higher inter-rater reliability of BEWE is likely related to index design. BEWE relies on broader categorical thresholds and records only the most severely affected surface per sextant, which may reduce interpretative ambiguity and promote more consistent scoring. The higher inter-rater reliability observed for BEWE compared with TWES 2.0 is consistent with previous clinical validation studies, which have highlighted the simplicity of the BEWE scoring system as a key factor contributing to its reproducibility and examiner agreement [12]. Previous studies have emphasized that the simplified categorical structure of BEWE supports consistent scoring and facilitates reliable use in routine clinical settings, which may explain its higher inter-rater reliability compared with more detailed indices [18,22]. In contrast, TWES 2.0 provides a more detailed, surface-specific assessment, increasing diagnostic resolution but also examiner dependence, particularly when subtle wear changes are evaluated.

Greater variability was observed in posterior sextants, especially on occlusal surfaces, which may reflect anatomical complexity and less clearly defined transitions between wear grades. Greater variability observed on posterior and palatal surfaces has also been reported in previous studies and is commonly attributed to anatomical complexity, limited visual cues, and less distinct transitions between wear grades in these regions [8,15,16,23,24,26]. Differences between raters were generally small and confined to adjacent score categories, suggesting inherent subjectivity of ordinal grading rather than systematic calibration bias. The absence of color information in intraoral scans may have contributed to slightly lower agreement for TWES 2.0.

TWES 2.0 scores were generally concentrated at the lower end of the scale (score 1), consistent with mild to moderate tooth wear in this adult population. Small but statistically significant differences between raters were observed in most sextants, with Rater 1 generally assigning slightly higher scores. These observations reflect minor inter-rater variability in grading subtle changes, particularly on posterior and palatal surfaces, without evidence of systematic bias.

When directly compared, BEWE demonstrated consistently higher inter-examiner reliability than the TWES 2.0 Screening Module across all sextants, as reflected by higher weighted kappa estimates. TWES 2.0 showed moderate to good reliability, with greater variability between examiners, particularly on posterior and palatal surfaces. These differences likely reflect the more detailed and surface-specific scoring structure of TWES 2.0, which may increase sensitivity to subtle wear features but also examiner dependence.

The observed reliability of BEWE and TWES 2.0 underscores their utility as practical screening tools in both routine dental practice and large-scale epidemiological studies. BEWE’s simplicity and ease of use make it particularly suitable for rapid risk assessment of dental erosion, whereas TWES 2.0 allows a more detailed evaluation of wear severity and differentiation of etiological mechanisms (erosion, attrition, abrasion). The combination of these indices can guide treatment planning and preventive strategies, enabling early identification and management of patients at risk of progressive tooth wear.

Importantly, although statistically significant inter-rater differences were observed, most disagreements involved differences of only one score category between examiners (e.g., score 1 vs. score 2), while larger discrepancies were uncommon. From a clinical perspective, such differences are unlikely to result in changes in BEWE cumulative risk category classification, which forms the basis for clinical decision-making, including preventive counseling, monitoring intervals, and referral for restorative intervention.

Consequently, the observed level of inter-examiner variability is not expected to alter treatment thresholds or patient management strategies in routine practice. BEWE may therefore be reliably used as a primary screening tool to stratify patients according to erosion risk and determine appropriate recall intervals, while TWES 2.0 can complement this approach by providing more detailed, surface-specific information to support individualized treatment planning and longitudinal monitoring.

Several limitations should be considered when interpreting the present findings. This study was retrospective and relied on anonymized intraoral scans, which limited the availability of patient-level data such as age beyond adulthood (≥18 years), dietary habits, oral hygiene behaviors, and parafunctional activity. In addition, sex information was unavailable for approximately 20% of the sample due to data anonymization.

Missing sextant-level data resulted from localized scan limitations, including incomplete capture, artifacts, or extensive restorative coverage preventing reliable scoring. Only paired sextant observations assessed by both examiners were included in the reliability analyses, leading to minor variations in sample size across sextants.

Minor differences in the number of paired observations between BEWE and TWES 2.0 resulted from differences in the scoring requirements of the two indices. BEWE records the most severely affected surface within each sextant using broader criteria, whereas TWES 2.0 requires more surface-specific interpretation, including distinctions related to dentine exposure and crown-height loss. Therefore, in some sextants, a score could be assigned confidently using BEWE, but the same sextant was excluded from TWES 2.0 analysis because the available scan information was insufficient for reliable TWES grading. Only paired observations scored by both examiners were included in each index-specific analysis.

All assessments were performed using digital intraoral scan–derived models without color or texture information. Although identical software environments were used by both examiners, the absence of optical cues inherent to clinical examination may have influenced scoring, particularly for more detailed indices such as TWES 2.0.

This study evaluated inter-rater reliability only and did not assess diagnostic validity against a clinical reference standard. Future studies should therefore incorporate a reference standard to evaluate both the reliability and diagnostic validity of these indices in digital workflows.

Potential variability related to the use of different intraoral scanner systems cannot be completely excluded. This should nevertheless be considered when interpreting the findings. Future studies should investigate the potential impact of different scanning systems on the reproducibility of tooth wear indices.

Future studies should aim to validate the application of BEWE and TWES 2.0 in prospective clinical settings, combining intraoral scan–based assessment with direct chairside examination. Longitudinal studies using repeated intraoral scans would be particularly valuable to evaluate the ability of these indices to monitor tooth wear progression over time.

Further research should investigate the potential impact of different intraoral scanner systems on the reproducibility of tooth wear indices. In particular, comparative analyses across scanners with different mesh resolutions and acquisition technologies may help to determine whether observed differences in reliability are primarily related to index design or to scanner-dependent differences geometric representation. Finally, the integration of automated or artificial intelligence–based wear detection tools may enhance objectivity and efficiency, potentially complementing established screening indices in digital workflows.

## 5. Conclusions

BEWE and TWES 2.0 are reliable and practical tools for the assessment of noncarious tooth wear. Inter-rater reliability was generally good, as demonstrated primarily by weighted kappa coefficients. While BEWE demonstrated slightly higher consistency across sextants, TWES 2.0 allows more detailed evaluation of wear severity, particularly on occlusal and palatal surfaces. These findings support the use of both indices in clinical practice and research for standardized monitoring, early detection, and risk assessment of tooth wear. Examiner calibration remains critical to ensure reproducibility, especially when using TWES 2.0.

## Supporting information

S1 Dataset Anonymized sextant-level BEWE and TWES 2.0 scores used for inter-rater reliability analysis. The dataset includes paired scores from both examiners for each sextant and was used for all statistical analyses reported in this study.
(XLSX)
